# Modulation of Acid Sphingomyelinase in Melanoma Reprogrammes the Tumour Immune Microenvironment

**DOI:** 10.1155/2015/370482

**Published:** 2015-05-26

**Authors:** Emma Assi, Davide Cervia, Laura Bizzozero, Annalisa Capobianco, Sarah Pambianco, Federica Morisi, Clara De Palma, Claudia Moscheni, Paolo Pellegrino, Emilio Clementi, Cristiana Perrotta

**Affiliations:** ^1^Scientific Institute IRCCS Eugenio Medea, 23842 Bosisio Parini, Italy; ^2^Division of Molecular Oncology, San Raffaele Scientific Institute, 20132 Milan, Italy; ^3^Unit of Clinical Pharmacology, National Research Council-Institute of Neuroscience, Department of Biomedical and Clinical Sciences “Luigi Sacco” (DIBIC), University Hospital “Luigi Sacco”, Università di Milano, 20157 Milan, Italy; ^4^Department for Innovation in Biological, Agro-Food and Forest Systems (DIBAF), Università della Tuscia, 01100 Viterbo, Italy; ^5^Department of Oncology, Università di Torino and Laboratory of Neurovascular Biology, Candiolo Cancer Institute, 10060 Candiolo, Italy; ^6^Division of Regenerative Medicine, San Raffaele Scientific Institute, 20132 Milan, Italy; ^7^Unit of Morphology, Department of Biomedical and Clinical Sciences “Luigi Sacco” (DIBIC), Università di Milano, 20157 Milan, Italy

## Abstract

The inflammatory microenvironment induces tumours to acquire an aggressive and immunosuppressive behaviour. Since acid sphingomyelinase (A-SMase) downregulation in melanoma was shown to determine a malignant phenotype, we aimed here to elucidate the role of A-SMase in the regulation of tumour immunogenic microenvironment using *in vivo* melanoma models in which A-SMase was either downregulated or maintained at constitutively high levels. We found high levels of inflammatory factors in low A-SMase expressing tumours, which also displayed an immunosuppressive/protumoural microenvironment: high levels of myeloid-derived suppressor cells (MDSCs) and regulatory T lymphocytes (Tregs), as well as low levels of dendritic cells (DCs). In contrast, the restoration of A-SMase in melanoma cells not only reduced tumour growth and immunosuppression, but also induced a high recruitment at tumour site of effector immune cells with an antitumoural function. Indeed, we observed a poor homing of MDSCs and Tregs and the increased recruitment of CD8^+^ and CD4^+^ T lymphocytes as well as the infiltration of DCs and CD8^+^/CD44^high^ T lymphocytes. This study demonstrates that change of A-SMase expression in cancer cells is sufficient *per se* to tune *in vivo* melanoma growth and that A-SMase levels modulate immune cells at tumour site. This may be taken into consideration in the setting of therapeutic strategies.

## 1. Introduction

Malignant melanoma is a highly metastatic skin cancer characterized by rapid growth, early metastasis, and resistance to chemotherapy and radiotherapy [1]. In the past the high immunogenic capacity of melanoma has fostered the development of immunotherapeutic strategies based on the stimulation of adaptive immunity T cell mediated against specific tumour antigen [2]. However, despite initial promising studies, the overall outcomes of the clinical trials based on immunotherapy are still far from being satisfying [3]. The lack of an effective immune reactivity to tumours may be explained by the protracted inflammation that occurs in melanoma microenvironment, eventually resulting in immunosuppression and thus in the failure of immune cells to reject the tumour [4, 5]. The inflammatory microenvironment in the tumour mass is the result of the secretion of inflammatory cytokines by both melanoma and stromal cells [6, 7]. It induces cancer cells to acquire a more aggressive phenotype and allows the recruitment in the tumour lesion of different immunosuppressive cells, such as myeloid-derived suppressor cells (MDSCs) and, consequently, regulatory T lymphocytes (Tregs) [5]. Various strategies have been used to decrease MDSC amounts and immunosuppressive functions in tumours, including melanomas [4, 8, 9]. However, the molecular mechanisms leading to the control of intratumoural MDSCs are poorly understood. The elucidation of these aspects may be crucial for the development of new approaches in melanoma treatment aimed at reducing MDSC-mediated immunosuppression.

In the last decades, the proapoptotic second messenger ceramide has drawn the attention as a fundamental player in cancer biology [10, 11]. Ceramide levels are significantly decreased in several tumours including melanomas, colon cancers, ovarian cancers, and gliomas; interestingly an inverse relationship has been found between ceramide levels and the stratification of tumours with high- and low-grade gliomas containing low and high levels of ceramide, respectively [12–14]. Of interest it seems particularly clear that cancer cells have evolved complex mechanisms to reduce ceramide levels, possibly through the modulation of their generating enzymes [15–17]. An example of this event is the spontaneous downregulation of the ceramide-generating enzyme acid sphingomyelinase (A-SMase) in melanoma cells during tumour progression that accounts for a more aggressive behaviour of melanomas in terms of tumour growth and metastatic ability [18].

This work has been designed to elucidate the possible role of A-SMase in determining melanoma immunogenic capacity with a specific attention on the modulation of tumour microenvironment. To this end, we analysed the immune cell infiltration in a mouse model of melanoma syngeneic transplant that is particularly aggressive and, more importantly, that recapitulates the downregulation of A-SMase during tumour progression [18]. A clone in which A-SMase is maintained at constitutively high levels during melanoma progression was analysed as well. We found that the progressive loss of A-SMase enhanced the immunosuppressive capacity of melanoma cells through the recruitment of MDSCs and Tregs at tumour site and the impairment of dendritic cells (DCs) maturation. Of importance, the upregulation of A-SMase enhanced the function of immune effector cells as DCs and cytotoxic T lymphocytes. This A-SMase-dependent modulation of microenvironment events may have a therapeutic outcome in terms of tumour growth.

## 2. Methods

### 2.1. Cell Models and Animals

The cell clones used in our experiments were generated from the murine melanoma cell line B16-F1 of American Type Culture Collection (LGC Standards, Sesto San Giovanni, Italy). As described in Bizzozero et al. [18], the low expressing A-SMase B16-W6_pSIL10 clone was generated from a subclone of the parental cell line B16-F1 (i.e., B16-W6) by transfecting the cells with the plasmid p*Silencer*4.1-CMV (Invitrogen-Life Technologies, Monza, Italy) harbouring a shRNA containing a sequence which gave the A-SMase silencing. The control clone used in our experiments (B16-pSILscr) was generated by transfecting B16-W6 cells with the plasmid p*Silencer*4.1-CMV harbouring a shRNA containing a scrambled sequence. The B16-B1A clone overexpressing A-SMase was generated as follows: the pEF1/Myc plasmid (Invitrogen-Life Technologies) containing cDNA for A-SMase [18] was transfected in B16-F1 cells using the Fugene transfection reagent (Promega, Milano, Italy), according to the manufacturer's protocol. Approximately 15 colonies resistant to the antibiotic G418 (500 *μ*g/mL) were tested for A-SMase expression and activity (see Section 3.3). A B16-F1 clone transfected with the empty vector pEF1/Myc (B16-pEF1) was used as control. Cells were routinely tested for* Mycoplasma* using a BioWhittaker MycoAlert* Mycoplasma* Detection Kit (Lonza Group, Basel, Switzerland). Cells were cultured in Iscove's modified Dulbecco's medium supplemented with 10% heat-inactivated foetal bovine serum (FBS), glutamine (200 mM), and penicillin/streptomycin (100 U/mL) and grown at 37°C in a humidified atmosphere containing 5% CO_2_.

Female C57BL/6 mice (6–8 weeks old) were purchased from Charles River Laboratories (Calco, Italy), housed in a regulated environment (23 ± 1°C, 50 ± 5% humidity) with a 12 h light/dark cycle (lights on at 08.00 a.m.), and provided with food and water* ad libitum*. All studies were conducted in accordance with the Italian law on animal care N° 116/1992 and the European Communities Council Directive EEC/609/86. The experimental protocols were approved by the Ethics Committee of the University of Milan. All efforts were made to reduce both animal suffering and the number of animals used.

### 2.2. Animal Handling and Tumour Growth

Mice received* s.c*. injections of 5 × 10^4^ cells in the lower-right flank on day 0 (ten animals per group at minimum) [18, 19]. Tumour growth was monitored every 2-3 days by means of external calliper measurements and volume calculation (length × width^2^/2), until mice reached IACUC euthanasia criteria, as, for instance, clinical signs of tumour or when tumour size exceeded 10% of body weight [19]. For PCR and flow cytometry analysis tumours were resected when they reached the volume of ca. 500 mm^3^. Late stage melanomas were explanted at a volume of ca. 1500 mm^3^.

### 2.3. Quantitative Real Time-PCR (qPCR)

The analysis of mRNA expression was performed as previously described [18, 20–22]. Briefly, total RNA from resected tumours and* in vitro* B16 clones was extracted with the High Pure RNA Tissue Kit and the High Pure RNA Isolation Kit, respectively (Roche Applied Science, Mannheim, Germany), according to the manufacturer's protocol. First-strand cDNA was generated from 1 *μ*g of total RNA using iScript Reverse Transcription Supermix (Bio-Rad, Hercules, CA, USA). As shown in Table 1, a set of primer pairs amplifying fragments ranging from 63 to 354 bp was designed to hybridise to unique regions of the appropriate gene sequence. qPCR was performed using SsoAdvanced Universal SYBR Green Supermix (Bio-Rad). The fold change was determined relative to the control after normalising to GAPDH (internal standard) through the use of the formula 2^−ΔΔCT^.

### 2.4. Flow Cytometry

After surgical excision, a single cell suspension from tumours was obtained by collagenase IV (0.2 mg/mL), dispase (2 mg/mL), and DNase I (0.1 mg/mL) treatment in Iscove's modified Dulbecco's medium, supplemented with 5% FBS, glutamine (200 mM), and penicillin/streptomycin (100 U/mL). After 30 min at 37°C in a shaking bath, the cell suspension was filtered (40 *μ*m pore-size membrane) and washed in phosphate buffer saline (PBS) added with 1% bovine serum albumin (BSA). Unspecific sites were blocked incubating samples for 20 min at room temperature in PBS containing 10% goat serum and 1% BSA. Cells were then incubated with fluorophore-conjugated antibodies for the staining of specific cellular markers (Table 2) in 1% BSA-PBS solution (1 h on ice). Cells were analysed by flow cytometry using a Gallios Flow Cytometer (Beckman-Coulter, Brea, CA, USA) and the software FCS Express 4 (De Novo System, Portland, OR, USA).

### 2.5. Western Blotting

The analysis of protein expression from* in vitro* B16 clones was performed as previously described [18, 20–23]. The rabbit polyclonal anti myc-tag (Cell Signaling Technology, Danvers, MA, USA) and the mouse monoclonal anti-TATA binding protein (TBP) (Abcam, Cambridge, UK) primary antibodies were used in combination with the appropriate secondary antibodies HRP-conjugated (Bio-Rad). Proteins were visualised using Bio-Rad Clarity Western ECL substrate with a Bio-Rad ChemiDoc MP imaging system. Bands were quantified for densitometry using the Bio-Rad Image Lab software.

### 2.6. A-SMase Activity

Cells (2 × 10^6^ cells/mL) were homogenised in the acid lysis buffer (50 mM sodium acetate, 1% Triton X-100, and 1 mM EDTA, pH 5) with freshly added protease inhibitor cocktail (cOmplete, Roche Applied Science) [24]. Sphingomyelinase activity was measured using the Amplex Red Sphingomyelinase Assay Kit (Molecular Probes-Life Technologies, Monza, Italy), as described in the manufacturer's protocol.

### 2.7. Apoptosis Detection

Apoptosis was measured by flow cytometry as described previously [18]. Briefly, adherent cells were detached using Enzyme-Free Cell Dissociation Buffer (Merck Millipore Billerica, MA, USA), washed twice with cold PBS, and incubated (1 × 10^6^ cells) with 5 *μ*L of FITC-labelled annexin V (Life Technologies, Monza, Italy) to detect phosphatidylserine exposure on the outer leaflet of the plasma membrane and 5 *μ*L of propidium iodide (PI) (eBioscience, San Diego, CA, USA) to exclude necrotic cells, in binding buffer (10 mM HEPES, 140 mM NaCl, and 2.5 mM CaCl_2_). After 15 min of incubation at room temperature, cells were acquired using a Gallios Flow Cytometer (Beckman-Coulter, Brea, CA, USA) and analysed by the software FCS Express 4 (De Novo System, Portland, OR, USA).

### 2.8. Statistical Analysis

Upon verification of normal distribution, statistical significance of raw data between the groups in each experiment was evaluated using unpaired Student's *t*-test (single comparisons) or one-way ANOVA followed by the Newman-Keuls posttest (multiple comparisons). Data belonging from different experiments were represented and averaged in the same graph. Tumour growth was analysed using two-way ANOVA, followed by the Bonferroni posttest. The GraphPad Prism software package (Graph Software, San Diego, CA, USA) was used. Results are expressed as means ± standard error of the mean (SEM) of the indicated *n* values.

### 2.9. Materials

Iscove's modified Dulbecco's medium, FBS, glutamine, penicillin/streptomycin, HEPES, G418, and PBS were purchased from Euroclone (Pero, Italy). Primer pairs were obtained from Primm Biotech (Milano, Italy). Dispase and DNase I were obtained from Gibco-Life Technologies (Monza, Italy) and Roche Applied Science, respectively. Goat serum was purchased from Vector Laboratories (Burlingame, CA, USA). Flow cytometry antibodies were purchased as follows: CD11b and Gr1 from Miltenyi Biotec, Bergisch Gladbach, Germany; CD11c, CD80, MHCII, CD4, CD8, CD25, CD44, and Foxp3 from eBioscience, San Diego, CA, USA. All other reagents were purchased from Sigma-Aldrich (Saint Louis, MO, USA).

## 3. Results

### 3.1. Downregulation of A-SMase Increases the Accumulation of MDSCs in Melanoma

We have previously demonstrated that during B16-F1 melanoma progression A-SMase is spontaneously downregulated thus conferring to melanoma cells a more aggressive phenotype [18]. To evaluate the impact of this event on the modulation of tumour microenvironment, mice were subcutaneously injected with B16-W6_pSIL10 cells, a loss-of-function model of A-SMase obtained by the knock-down of A-SMase in the parental cell line B16-F1 [18]; we then analysed the* in vivo* behaviour of the established skin melanoma in terms of cytokine expression and tumour-infiltrating immune cells. The parental cell line B16-pSILscr served as control.

**Figure 1:**
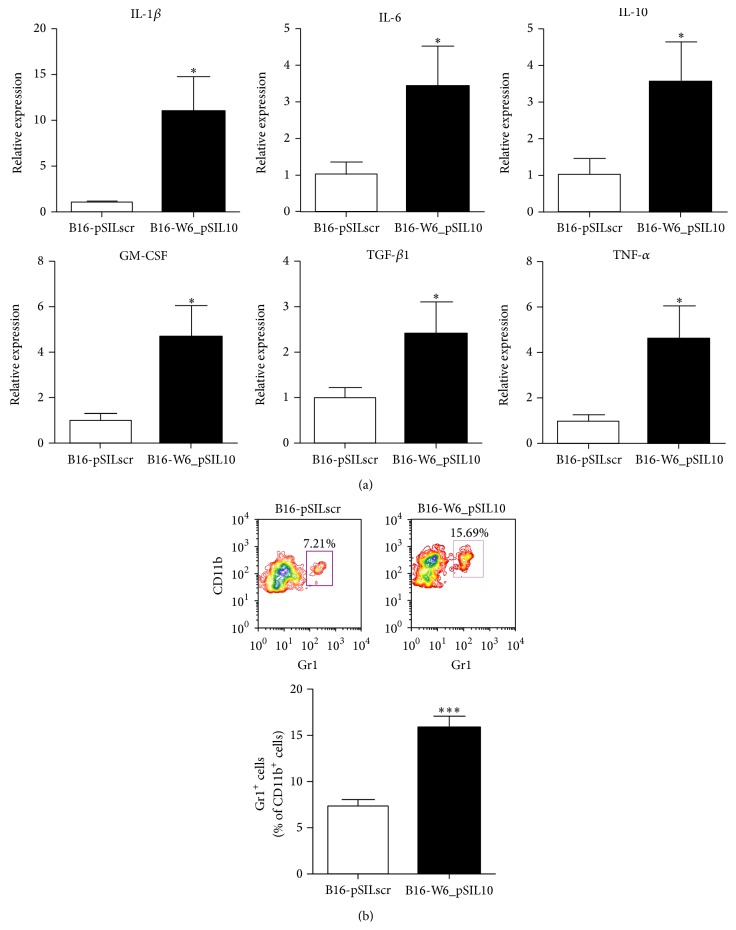
Downregulation of A-SMase increases the accumulation of MDSCs in melanoma. Tumours were resected when they reached the volume of ca. 500 mm^3^ and treated to extract mRNA or obtain a cell suspension. (a) qPCR analysis of mRNA of IL-1*β*, IL-6, IL-10, GM-CSF, TGF-*β*1, and TNF-*α*. The histograms are representative of results obtained from at least seven to twelve different animals per experimental group. (b) Flow cytometry analysis of tumour-infiltrating immune cells. The cell suspensions obtained from different tumours were stained with anti-CD11b-APC and anti-Gr1-FITC antibodies. A representative dot plot of CD11b^+^/Gr1^+^ cells in the CD11b^+^ population cell is shown (*upper panel*). The histograms represent the results obtained from at least ten to fifteen different animals per experimental group (*lower panel*). Values are expressed as mean ± SEM. ^*∗∗∗*^ *P* < 0.001 versus B16-pSILscr control.

The mRNA analysis by qPCR in the explanted melanomas of a panel of different inflammatory factors showed a significant accumulation at B16-W6_pSIL10 tumour site of interleukin- (IL-) 1*β*, IL-6, IL-10, granulocyte-macrophage colony-stimulating factor (GM-CSF), transforming growth factor- (TGF-) *β*1, and tumour necrosis factor- (TNF-) *α* (Figure 1(a)). A sustained secretion and maintenance of inflammatory mediators during tumour progression stimulate the enrichment and activation of MDSCs then leading to immunosuppression [4]. Thus, we evaluated the presence of MDSCs (CD11b^+^/Gr1^+^ cells) in the tumour mass by flow cytometry. As shown in Figure 1(b), we found a significant increase in CD11b^+^/Gr1^+^ cells infiltrating B16-W6_pSIL10 tumours with respect to control. Of notice, the percentage of MDSCs in B16-F1 tumours at a late stage of growth, in which A-SMase was spontaneously downregulated [18], was similar to that of B16-W6_pSIL10 tumours (Supplementary Figure 1(a) in Supplementary Material available online at http://dx.doi.org/10.1155/2015/370482). These observations support the role of A-SMase downregulation in favouring the recruitment of MDSCs at the tumour lesion.

### 3.2. A-SMase-Related MDSC Accumulation Determines an Immunosuppressive Microenvironment

**Figure 2:**
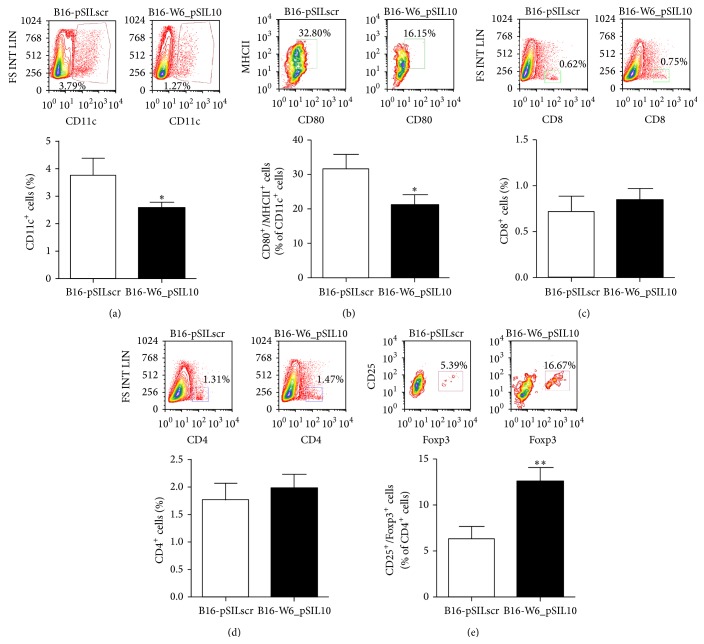
A-SMase induced MDSC accumulation determines an immunosuppressive microenvironment. Flow cytometry analysis of tumour-infiltrating immune cells. Tumour cell suspensions obtained from resected tumours were stained with the specific fluorescent conjugated antibodies for CD11c, CD80, MHCII, CD8, CD4, CD25, and Foxp3 antibodies. (a) A representative dot plot of gated CD11c^+^ cells is shown (*upper panel*). The histograms represent the data obtained from ten to fifteen animals per experimental group (*lower panel*). (b) A representative dot plot of CD80^+^/MHCII^+^ cells in the CD11c^+^ cell population is shown (*upper panel*). The histograms represent the data obtained from ten to fifteen animals per experimental group (*lower panel*). (c-d) A representative dot plot of CD8^+^ cells (c) and CD4^+^ cells (d) is shown (*upper panel*). The histograms represent the data obtained from ten to fifteen animals per experimental group (*lower panel*). (e) A representative dot plot of CD25^+^/Foxp3^+^ in the CD4^+^ cell population is shown (*upper panel*). The histograms represent the data obtained from ten to fifteen animals per experimental group (*lower panel*). Values in each histogram are expressed as mean ± SEM. ^*∗*^ *P* < 0.05; ^*∗∗*^ *P* < 0.01 versus B16-pSILscr control.

MDSCs exert their immunosuppressive function through the impairment of DCs (CD11c^+^ cells) and cytotoxic T lymphocytes (CD8^+^ cells) [25–27] and the recruitment of Tregs (CD4^+^/CD25^+^/Foxp3^+^ cells) [40]. Flow cytometry analysis of immune cells from explanted melanomas showed a significant decrease of CD11c^+^ cells at B16-W6_pSIL10 tumour site when compared to control (Figure 2(a)). Interestingly recruited DCs displayed an immature and anergic phenotype as indicated by the reduction of the costimulatory markers CD80 and MHCII (Figure 2(b)). Although we did not observe a difference in CD8^+^ and CD4^+^ T infiltrating lymphocytes (Figures 2(c) and 2(d)), we found a significant increase in the number of Tregs in B16-W6_pSIL10 tumours with respect to control (Figure 2(e)). Similar results were obtained in B16-F1 melanoma at late stage of growth (Supplementary Figure 1(b)). Taken together, these results indicate that a low expression of A-SMase in melanoma cells induces an immunosuppressive tumour microenvironment.

### 3.3. Restoring A-SMase Expression Switches the Tumour Microenvironment from a Pro- to an Antitumoural Phenotype and Reduces Melanoma Growth

**Figure 3:**
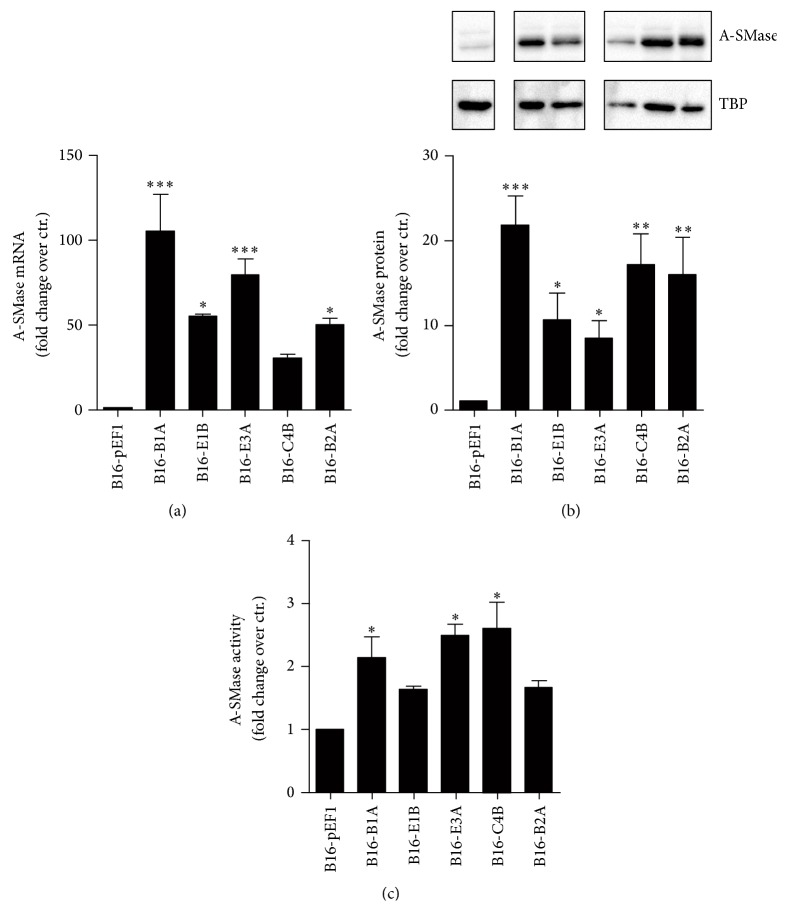
Generation of B16-F1 clones stably expressed A-SMase. Evaluation of A-SMase expression/activity in B16-F1-derived clones overexpressing A-SMase. The B16-pEF1 clone transfected with the empty vector (pEF1) was used as control. (a) qPCR analysis of A-SMase expression. (b) Western blotting analysis of A-SMase. Image is representative of at least three independent experiments. TBP was used as loading control. The histograms represent the densitometric values normalized on TBP. (c) A-SMase activity on cell lysates measured as sphingomyelin hydrolysis at pH 5.5. Values are expressed as fold increases over control ± SEM (*n* = 3). ^*∗*^ *P* < 0.05; ^*∗∗*^ *P* < 0.01;^*∗∗∗*^ *P* < 0.001 versus B16-pEF1 control.

**Figure 4:**
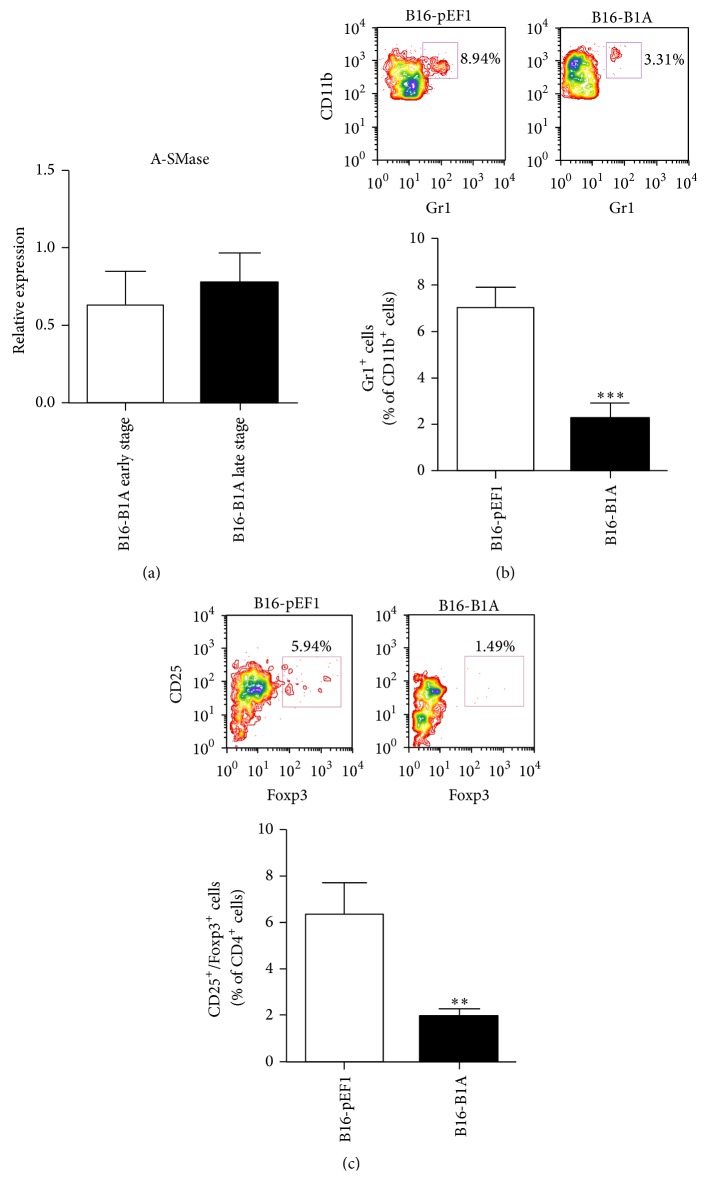
A-SMase expression reprogrammes the tumour microenvironment. (a) Analysis of A-SMase expression in B1A tumours* in vivo*. Tumours were resected when they reached the volume of ca. 500 mm^3^ (early stage) or 1500 mm^3^. A-SMase expression was evaluated by qPCR. The histograms represent the data obtained from five to seven animals per experimental group. (b-c) Flow cytometry analysis of tumour-infiltrating immune cells. Tumour cell suspensions obtained from resected tumours were stained with the specific fluorescent conjugated antibodies for CD11b, Gr1, CD4, CD25, and Foxp3 antibodies. (b) A representative dot plot of CD11b^+^/Gr1^+^ cells in the CD11b^+^ population cell is shown (*upper panel*). The histograms represent the data obtained from ten to fifteen animals per experimental group (*lower panel*). (c) A representative dot plot of CD25^+^/Foxp3^+^ in the CD4^+^ cell population is shown (*upper panel*). The histograms represent the data obtained from ten to fifteen animals per experimental group (*lower panel*). Values in each histogram are expressed as mean ± SEM. ^*∗∗*^ *P* < 0.01 versus B16-pEF1 control.

**Figure 5:**
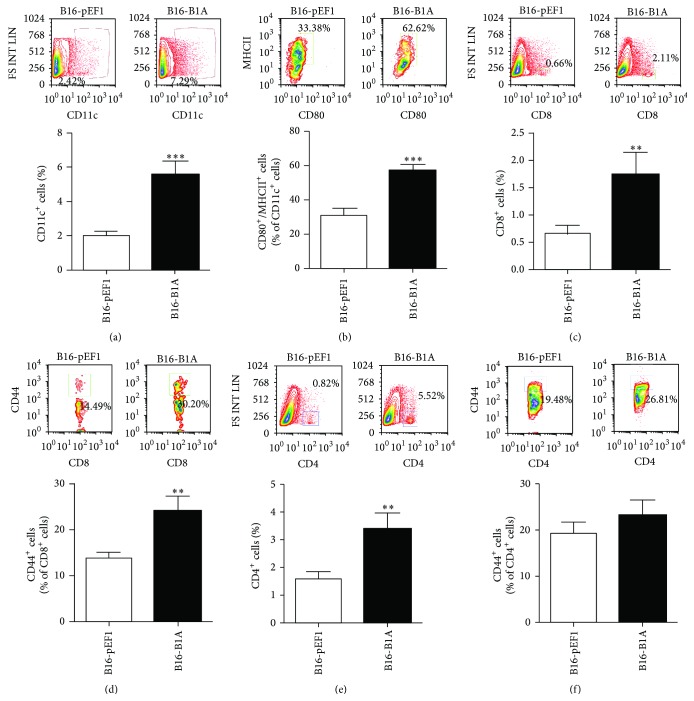
A-SMase expression increases the recruitment of effector antitumoural immune cells. Flow cytometry analysis of tumour-infiltrating immune cells. Tumour cell suspensions obtained from resected tumours were stained with the specific fluorescent conjugated antibodies for CD11c, CD80, MHCII, CD8, CD4, and CD44 antibodies. (a) A representative dot plot of gated CD11c^+^ cells is shown (*upper panel*). The histograms represent the data obtained from ten to fifteen animals per experimental group (*lower panel*). (b) A representative dot plot of CD80^+^/MHCII^+^ cells in the CD11c^+^ cell population is shown (*upper panel*). The histograms represent the data obtained from ten to fifteen animals per experimental group (*lower panel*). (c) A representative dot plot of CD8^+^ cells is shown (*upper panel*). The histograms represent the data obtained from ten to fifteen animals per experimental group (*lower panel*). (d) A representative dot plot of CD8^+^/CD44^+^ in the CD8^+^ cell population is shown (*upper panel*). The histograms represent the data obtained from ten to fifteen animals per experimental group (*lower panel*). (e) A representative dot plot of CD4^+^ cells is shown (*upper panel*). (f) A representative dot plot of CD4^+^/CD44^+^ in the CD4^+^ cell population is shown (*upper panel*). The histograms represent the data obtained from ten to fifteen animals per experimental group (*lower panel*). Values in each histogram are expressed as mean ± SEM. ^*∗*^ *P* < 0.05; ^*∗∗*^ *P* < 0.01 versus B16-pEF1 control.

To demonstrate unambiguously that A-SMase expression by melanoma cells modulates tumour microenvironment, different gain-of-function models of A-SMase were generated by stable transfection of A-SMase in B16-F1 cells. Measurements of A-SMase mRNA (Figure 3(a)), protein expression (Figure 3(b)), and activity (Figure 3(c)) in these A-SMase transfected clones with respect to parental control (B16-pEF1) allowed us to select B16-B1A as the cells overexpressing A-SMase to be used for* in vivo* studies as follows. In particular, mice were subcutaneously injected with B16-B1A cells and the explanted melanomas analysed for A-SMase expression and the immune cell composition. The cell clone B16-pEF1 served as control. As expected, B16-B1A tumour maintained stable levels of A-SMase mRNA levels during growth (Figure 4(a)). Of interest, the number of MDSCs and Tregs decreased significantly in B16-B1A tumour when compared to control (Figures 4(b) and 4(c)). This event was paralleled by the increase of DCs infiltrate (Figure 5(a)). These cells partially recovered their maturation status as demonstrated by the increase in expression of the costimulatory markers CD80 and MHCII (Figure 5(b)). Unexpectedly, even CD8^+^ and CD4^+^ T infiltrating lymphocytes as well as the CD8^+^/CD44^high^ activated T lymphocytes were significantly higher in B16-B1A tumours with respect to B16-pEF1 ones (Figures 5(c)–5(e)); no differences were observed in the population of CD4^+^/CD44^high^ T lymphocytes (Figure 5(f)). These data indicate that A-SMase restoration in melanoma cells results in the establishment of an antitumoural microenvironment through both the reduction of protumoural immune cells and the recruitment of antitumoural immune cells at tumour site.

**Figure 6:**
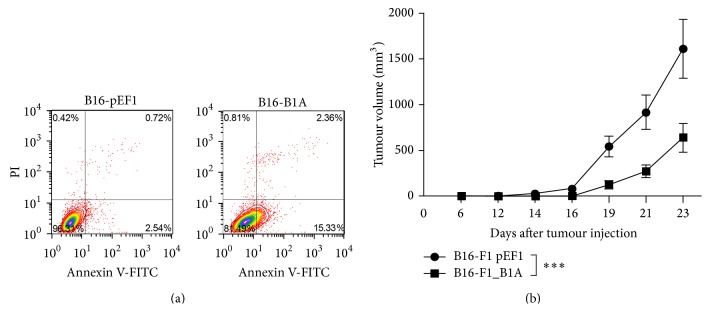
A-SMase expression reduces tumour growth. (a) Flow cytometry analysis of cell death of B16-pEF1 and B16-B1A cells. Apoptosis/necrosis were evaluated by the double staining with annexin V-FITC and PI. A representative dot plot for each cell clone is shown (*n* = 3). (b) Tumour growth analysis. BC57BL/6 mice (10 animals per group) were injected in the right flank with B16-pEF1 and B16-B1A (5 × 10^4^ cells). Tumour growth was monitored by measuring tumour volume (mm^3^) every 2-3 days. Values are expressed as mean ± SEM. ^*∗∗∗*^ *P* < 0.001.

Finally we addressed whether the overexpression of A-SMase may have therapeutic efficacy. The analysis of melanoma apoptosis* in vitro* revealed that the higher levels of A-SMase in B16-B1A correlate with an increase of apoptotic cell number when compared to the B16-pEF1 control cells (Figure 6(a)), thus confirming the fundamental role played by A-SMase in sensitivity to apoptosis. Of notice, as shown in the growth analysis* in vivo* of Figure 6, we observed a significant delay in the outgrowth of B16-B1A tumours* versus* control. These data are in line with our previous results in which the silencing of A-SMase resulted in an increase in melanoma growth [18] and confirms the inverse correlation between A-SMase expression and melanoma progression.

## 4. Discussion

The role of A-SMase in the response of tumours to chemotherapy and radiotherapy is a well-established concept [14]. Indeed, the enzyme contributes significantly to the cytotoxic effects of several anticancer drugs such as cisplatin, retinoids, and doxorubicin [28–32]. Moreover it has been demonstrated that the peritumoural injection of recombinant A-SMase sensitises mouse subcutaneous melanomas to the antineoplastic effects of radiotherapy [33]. Recently, A-SMase downregulation was shown to favour human and mouse melanoma ability to grow, invade, and metastasise [18]. We now show that change of A-SMase expression in cancer cells is sufficient* per se* to tune* in vivo* melanoma growth. In addition, our data indicated that A-SMase levels modulate immune cells at tumour site, thus suggesting the role of A-SMase as an immune-regulating factor of melanoma tumour microenvironment.

It is well known that the interactions between immune system components recruited into the tumour microenvironment are crucial for tumour development and progression, relying on inflammatory factors and cells that contribute to cell transformation, support cancerous cell survival, resist immunological destruction, and facilitate invasion and metastasis [6, 34]. In keeping with the notion that the protumoural microenvironment can be considered the product of a developing cross talk between cancerous cells and stromal cells, here we demonstrate a close correlation between the extent and type of tumour infiltrate and A-SMase levels. Specifically, low A-SMase in melanoma accounts for the high expression of factors involved in inflammation (i.e., IL-1*β*, IL-6, GM-CSF, and TNF-*α*), which have been positively associated with cancer onset and progression [6, 34]. In contrast with the A-SMase-mediated inhibitory effect on proinflammatory cytokines we also found that low A-SMase in melanoma accounts for the high expression of the anti-inflammatory cytokines IL-10 and TGF-*β*1. Of interest, both cytokines are generally accepted as major immunosuppressive cytokines expressed in tumours including melanoma, although there is still a debate on their effective role in antitumour immune response [6, 34–38]. Our data indicate that A-SMase decrease in melanoma accounts for the establishment of a high immunosuppressive and protumoural microenvironment. In this respect, tumours with low A-SMase levels, that is, B16-F1_psil10 and B16-F1 at late stage of development, display an increase of MDSCs infiltration, already known to be responsible for the establishment of immunosuppression [5, 39]. MDSCs accumulation in the tumour mass determines the impairment of DCs and cytotoxic T lymphocytes [25, 27] and the recruitment of regulatory Tregs [40]. In this line, in low A-SMase expressing tumours we found low levels of DCs; of interest these cells also showed an immature and anergic phenotype, a typical feature of melanoma infiltrating DCs resulting in the inability to trigger the activation of tumour specific CD8^+^ and CD4^+^ T lymphocytes [41, 42]. Moreover low A-SMase expressing tumours displayed increased Treg infiltration which is often associated with poor clinical outcome and tumour progression in different cancers [43]. This led to the hypothesis that the naturally occurring A-SMase decrease in melanoma cells during tumour progression contributes to the induction of immune tolerance and immunosuppression in tumour microenvironment and eventually in the acquisition of the high aggressive behaviour of melanoma* in vivo* [18].

In support of this hypothesis, the restoration of A-SMase expression in melanoma cells not only reduces tumour growth and immunosuppression, but also accounts for a high recruitment in the tumour microenvironment of effector immune cells with an antitumoural function. Indeed, we observed a poor homing of MDSCs and Tregs in tumours in which a sustained expression of A-SMase is maintained during their progression, that is, B16-B1A. As a consequence, we observed the increased recruitment of CD8^+^ and CD4^+^ T lymphocytes at the tumour lesion and especially the infiltration of mature DCs and CD8^+^/CD44^high^ memory/activated T lymphocytes [44–46]. The accumulation of effector immune cells such as DCs and CD8^+^ T lymphocytes into primary tumour lesions is associated with prolonged survival and a reduced incidence of metastases in patients with several types of solid cancer, including primary cutaneous melanomas [47, 48]. Based on this evidence, the current preclinical and clinical studies regarding melanoma immunotherapy are centred on the administration of DCs or CD8^+^ cells in the form of DC vaccines or adoptive T cell transfer, preceded by radiotherapy or chemotherapy to induce lymphodepletion and the ensuing elimination of immunosuppressive MDSCs and Tregs [49–52]. Consistent with these reports, our findings on the role of A-SMase upregulation in reprogramming the status of tumour microenvironment open new vistas in therapeutic perspective. In particular, the possibility that A-SMase overexpression “educates” tumour microenvironment against cancer cells encourages the use of therapeutic approaches to increase the enzyme expression/activity. This would create an antitumoural microenvironment suitable either for triggering an appropriate adaptive immune response against melanoma or for improving the efficacy of other immunotherapy strategies. In this respect, restoring A-SMase in melanomas by genetic overexpression or by recombinant protein administration, a protocol currently under examination for Niemann-Pick type B patients [53], might be considered a useful adjuvant for cancer therapy.

## 5. Conclusion

The evaluation of the role of A-SMase in many pathophysiological processes is a developing field [14, 28, 54–61]. This study demonstrates for the first time the central role of A-SMase expressed by melanoma cells in orchestrating the cross talk with the surrounding microenvironment. These interactions are crucial for tumour fate, lying on its rejection or progression. The discovery of this A-SMase function exerted on the tumour-infiltrating immune cells suggests that the antineoplastic effect of the enzyme goes beyond the well-established role in cancer cell death but involves a more complex network that may be taken into consideration in the setting of therapeutic strategies.

## Supplementary Material

B16-F1 tumours, in which A-SMase is spontaneously down regulated during melanoma progression [18], excised at late stage of growth (i.e. when they reached a volume of 1500 mm^3^) showed a strong increase of MDSCs and Treg cells. This is in line with the data obtained in B16-W6_pSIL10 tumours and supports the fundamental role of A-SMase in the recruitment of immune cells at the tumour lesion.

## Figures and Tables

**Table 1:** Primer pairs designed for PCR analysis.

Name	Symbol	Accession number	Primer sequence	Amplicon
IL-1*β*	*il1b *	NM_008361	F: 5′-GCAACTGTTCCTGAACTCAACT-3′ R: 5′-ATCTTTTGGGGTCCGTCAACT-3′	89 bp

IL-6	*il6 *	NM_031168	F: 5′-TAGTCCTTCCTACCCCAATTTCC-3′ R: 5′-TTGGTCCTTAGCCACTCCTTC-3′	76 bp

Il-10	*il10 *	NM_010548	F: 5′-GCTCTTACTGACTGGCATGAG-3′ R: 5′-CGCAGCTCTAGGAGCATGTG-3′	105 bp

GM-CSF	*csf2 *	NM_009969	F: 5′-GGCCTTGGAAGCATGTAGAGG-3′ R: 5′-GGAGAACTCGTTAGAGACGACTT-3′	104 bp

TGF-*β*1	*tgfb1 *	NM_011577	F: 5′-AAACGGAAGCGCATCGAA-3′ R: 5′-GGGACTGGCGAGCCTTAGTT-3′	63 bp

TNF-*α*	*tnf *	NM_013693	F: 5′-TTCTGTCTACTGAACTTCGGGGTGATCGGTCC-3′ R: 5′-GTATGAGATAGCAAATCGGCTGACGGTGTGGG-3′	354 bp

GAPDH	*gapdh *	NM_008084	F: 5′-ACCCAGAAGACTGTGGATGG-3′ R: 5′-ACACATTGGGGGTAGGAACA-3′	172 bp

A-SMase(v.1,2)	*smpd1 *	NM_000543; NM_001007593	F: 5′-TGGCTCTATGAAGCGATGGC-3′ R: 5′-TTGAGAGAGATGAGGCGGAGAC-3′	125 bp

F: forward; R: reverse.

**Table 2:** List of the antibodies used in flow cytometry for immune cells analysis.

Protein name	Conjugation	Clone
CD11b	APC	M1/70.15.11.5
Gr1	FITC	RB6-8C5
CD4	PE-Cy5	GK1.5
CD8	PE-Cy5	eBioH35-17.2
CD44	PE-Cy7	IM7
CD25	PE-Cy7	PC61.5
Foxp3	PE	150D/E4
CD11c	PE	N418
CD80	FITC	16-10A1
MHCII	PE-Cy5	M5/114.15.2
