# Supplementary material for: Modulation of Acid Sphingomyelinase in Melanoma Reprogrammes the Tumour Immune Microenvironment
Source: Mediators Inflamm. 2015 May 26;2015:370482. doi: 10.1155/2015/370482 (PMC4460251; doi:10.1155/2015/370482)
Supplement: Supplementary file 1 — B16-F1 tumours, in which A-SMase is spontaneously down regulated during melanoma progression [18], excised at late stage of growth (i.e. when they reached a volume of 1500 mm3) showed a strong increase of MDSCs and Treg cells. This is in line with the data obtained in B16-W6_pSIL10 tumours and supports the fundamental role of A-SMase in the recruitment of immune cells at the tumour lesion. [file 370482.f1.pdf]

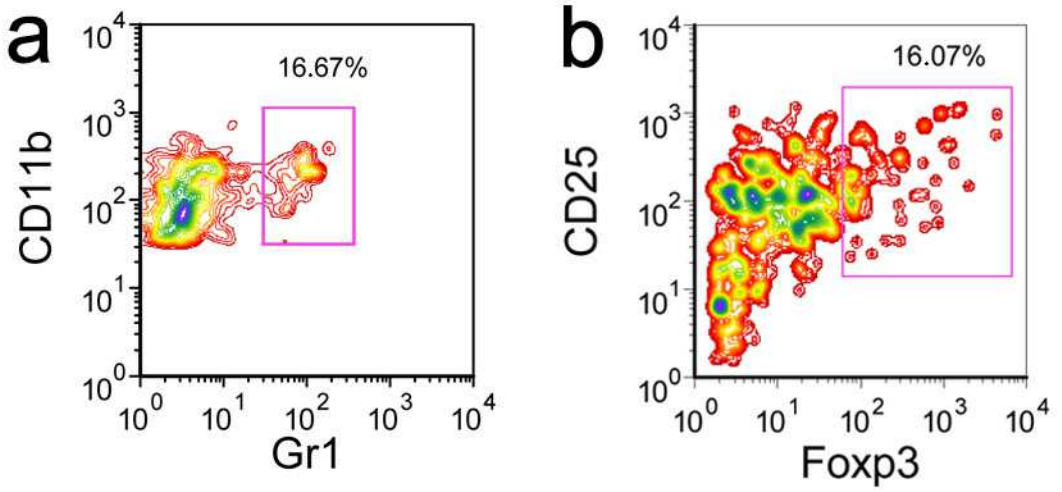

Suppl. Fig. 1

Supplementary figure 1: *MDSC and Treg recruitment in B16-F1 late stage tumours.*

(a-b) Tumour cell suspensions obtained from resected tumours were stained with the specific fluorescent conjugated antibodies for: CD11b, Gr1, CD4, CD25 and Foxp3 antibodies. (a) A representative dot plot of $CD11b^+/Gr1^+$ cells in the $CD11b^+$ population cell is shown (n = five animals per experimental group). (b) A representative dot plot of $CD25^+/Foxp3^+$ in the $CD4^+$ cell population is shown (n = five animals per experimental group).
